# Determinants and spatial distribution of early newborn care in Somalia: evidence from the 2020 Somalia health and demographic survey

**DOI:** 10.1016/j.jped.2025.101496

**Published:** 2026-01-06

**Authors:** Abdirahman Omer Ali, Awo Mohamed Kahie, Nura Mohamed Omer, Muhyadin Yusuf Dahir, Abdisalam Mahdi Hassan, Hodo Abdi Abdillahi, Md. Moyazzem Hossain

**Affiliations:** a Amoud University, School of Medicine and Surgery, College of Health Sciences, Borama, Somalia; b Amoud University, School of Postgraduate Studies and Research, Amoud Valley, Borama, Somalia; c Borama Regional Hospital, Pediatric Department, Borama, Somalia; d Jahangirnagar University, Department of Statistics and Data Science, Savar, Dhaka 1342, Bangladesh

**Keywords:** Early newborn care, Multilevel modeling, Somalia, Spatial analysis, Sustainable development goals (SDGs)

## Abstract

**Objective:**

Early newborn care (ENC) is important for reducing neonatal mortality; however, the rate of receiving adequate newborn care is low in Somalia. Therefore, this study aimed to identify the individual, household, and community-level determinants and map the spatial patterns of ENC in Somalia to take the required actions and policies.

**Methods:**

This study considered a weighted sample of 15,024 mother-newborn pairs extracted from a countrywide cross-sectional survey, the 2020 Somali Health and Demographic Survey (SHDS). Multilevel logistic regression was employed to identify factors associated with receiving adequate ENC within two days of birth. Global Moran’s I and Getis-Ord Gi* statistics were used for spatial analysis.

**Results:**

The prevalence of adequate ENC was critically low (5.23%). Findings revealed that delivery in a health facility (vs. home: Adjusted Odds Ratio (AOR) = 0.22, 95% CI: 0.18-0.26), higher household wealth (richest vs. poorest: AOR = 2.02, 95% CI: 1.59-2.57), and higher birth order were influential predictors of receiving ENC. However, having multiple or a higher number of living children was associated with significantly lower odds of receiving ENC. Spatial analysis identified a statistically significant hot spot of higher ENC coverage in the northwestern regions and cold spots of extremely low coverage in the south.

**Conclusion:**

In Somalia, early neonatal care is shockingly insufficient, and significant social and regional disparities. To achieve Sustainable Development Goal 3, interventions must be implemented based on identified cold spots, prioritizing the strengthening of access to skilled care at birth, and addressing the economic vulnerabilities of families.

## Introduction

Early newborn care (ENC), which comprises a series of essential interventions administered to a neonate typically within the initial 48 hours to seven days following birth, is critical for mitigating neonatal morbidity and mortality.^1^, ^2^, ^3^ This timeframe, frequently designated as early postnatal care (PNC), encompasses vital practices such as thermal care (including immediate drying, warming, and the postponement of bathing), hygienic management of the umbilical cord, prompt initiation and facilitation of exclusive breastfeeding, in addition to the timely identification and handling of danger signs and complications in newborns.^2^, ^3^, ^4^ The World Health Organization (WHO) advocates for all newborns to undergo a health assessment by a qualified provider within 24 hours post-delivery, followed by additional evaluations at predetermined intervals, to ensure well-being and furnish essential support for both the mother and the infant.^5^ Effective ENC not only addresses immediate survival requirements but also establishes a groundwork for long-term health and developmental outcomes, acting as a crucial metric for the quality and accessibility of maternal and child health services.^6^

The first days of life represent the most fragile window for human survival, a critical juncture where simple, low-cost interventions can mean the difference between life and death.^7^, ^8^, ^9^ On a global scale, millions of newborns fall victim to primarily preventable or treatable conditions, including complications arising from preterm birth, intrapartum-related incidents (such as birth asphyxia), and various infections.^10^, ^11^, ^12^ An alarmingly significant fraction of these deaths materializes during the first 24 hours and the subsequent week of life, highlighting the critical need for swift and effective early newborn care.^6^,^13^ In settings characterized by resource limitations, particularly those afflicted by conflict and instability, the vulnerability of newborns is significantly exacerbated, rendering the provision and acceptance of ENC more than merely a health intervention; it emerges as an essential obligation for the protection of future generations.^4^,^14^

Globally, notwithstanding significant progress in the reduction of under-five mortality rates, neonatal mortality has experienced a more measured decline, presently accounting for nearly half of all under-five fatalities.^11^,^15^ In the year 2020, it was estimated that 2.4 million newborns died worldwide, with Sub-Saharan Africa enduring the most substantial burden, exhibiting the highest neonatal mortality rates (NMR) globally, approximately 27 deaths for every 1,000 live births.^4^,^14^,^16^ Within this region, the Horn of Africa persistently encounters severe challenges. Somalia, a nation plagued by decades of conflict, political instability, recurrent climatic adversities (including droughts and floods), and widespread displacement, demonstrates one of the highest NMRs worldwide, with recent reports indicating figures significantly surpassing regional averages.^4^,^14^,^17^,^18^ Goal 3 of the UN Sustainable Development Goals aims to reduce avoidable infant and child mortality worldwide by 2030.^19^

The Somali Health and Demographic Survey (SHDS) conducted in 2020 revealed an infant mortality rate of 73 fatalities per 1,000 live births, with neonatal fatalities representing a significant proportion of this statistic.^18^ The country's health system is profoundly disjointed, characterized by a shortage of skilled health personnel, inadequate infrastructure development, and limited access to essential maternal and neonatal health services, particularly in rural and conflict-affected regions.^4^,^7^ Although investigations in the neighboring nation of Ethiopia have underscored low levels of essential newborn care (ENC) uptake alongside considerable geographic disparities.^1^,^6^,^7^,^20^ However, evidence emanating from Somalia, particularly recent national-level data concerning specific ENC practices, remains limited. Prior localized evaluations, such as those conducted in Bossaso, have signaled a variability in the quality and availability of essential newborn care.^4^ Notwithstanding the critical significance of ENC, there exists a substantial deficit of recent, nationally representative evidence concerning the determinants and, crucially, the geographic distribution of early newborn care practices within Somalia. While certain studies have addressed postnatal care in a general sense or concentrated on specific regions.^4^,^8^ None has employed the most recent 2020 SHDS dataset to perform a thorough spatial and multilevel analysis of ENC across the entirety of Somalia. This study aims to bridge the significant knowledge gap by identifying the individual, household, and community-level determinants that influence the uptake of key ENC components and mapping their geographic variations throughout Somalia. The authors believed that the findings of this research would equip policymakers, program implementers, and international collaborators with the rigorous evidence needed to develop contextually relevant and geographically focused interventions. The authors also suggest facilitating equal distribution of resources, strengthening maternal and newborn health initiatives, and accelerating progress towards reducing preventable neonatal deaths in Somalia to meet the Sustainable Development Goals related to health and well-being.

## Methods and materials

### Data source

This study utilized secondary data from the 2020 Somali Demographic and Health Survey (SDHS). The SDHS employed a multistage stratified cluster sampling method.^21^ A three-stage design was used for urban and rural areas, while a two-stage design was applied to nomadic regions. In total, 55 sampling strata were established, categorizing each region into urban, rural, and nomadic sectors, except for Banadir, which is entirely urban. Due to security issues, several areas were excluded: three strata from Lower Shabelle, three from Middle Juba, and two unspecified strata, leading to a final count of 47 sampling strata.^22^ After deleting the incomplete cases, this study included a sample of 15,024 respondents from the 2019 Somali Demographic and Health Survey (SDHS. This sample was representative of 16 geographic regions in Somalia, encompassing urban, rural, and nomadic areas, as well as various types of residences.

### Outcome variable

The dependent variable in this study is defined as “Newborn care practice within two days of the postpartum period,” measured as a “Yes/No”. It consists of five components: cord examination, temperature measurement, counseling on danger signs, breastfeeding guidance, and observation of breastfeeding. A total score is calculated based on responses to the five components as:

Score = Cord Exam + Temp Measurement + Danger Signs Counseling + BF Guidance + BF Observation.

The total score ranges from 0 to 5 and then the outcome variable is dichotomized into two groups: participants whose newborns lack one of the five components are categorized as not receiving adequate newborn care, while those who meet all five components are considered to have received proper care, i.e., if Score = 5, the outcome variable = 1 and consider “Yes” that means adequate care), otherwise, the authors consider the outcome variable = 0 indicating “No” that means inadequate care.

### Covariates

Various demographic and social factors, including region, residence type, maternal education, media consumption, marital status, birth type, child sex, wealth index, maternal age, birth order, women’s occupation, barriers to care, place of delivery, household size, and the number of children, were considered as covariates. The list used in this study is presented in Supplementary Table 1.

### Choropleth map

The choropleth map is a popular quantitative thematic map form where fill colors, tints, or patterns are used to depict the magnitudes of area-based statistics (often derived attribute data) as they occur within the unit area borders.^23^ This map offers a clear visual representation of geographical patterns, enabling readers to quickly identify areas where values are high, low, or clustered, and highlighting regional disparities, hotspots, and areas that require policy attention. The choropleth map visually represents the spatial distribution of immediate newborn care in Somalia. It utilizes color gradients to indicate varying levels of care across different regions, facilitating an easy comparison of practices. Darker shades typically signify higher levels of newborn care, while lighter shades indicate lower levels.

### Spatial autocorrelation analysis

Spatial autocorrelation (Global Moran’s I and local Moran's I) assesses the correlation between a variable and its surrounding values, measuring the overall spatial autocorrelation of newborn care practices. A value close to -1 indicates strong negative spatial autocorrelation, suggesting dispersion, while a value near +1 indicates strong positive spatial autocorrelation, suggesting clustering. A value around zero implies random distribution, indicating no spatial autocorrelation. A statistically significant Moran’s I (p < 0.05) rejects the null hypothesis that newborn care is randomly distributed, confirming the presence of spatial autocorrelation. Additionally, hot spot analysis using Getis-Ord Gi* statistics evaluates how spatial autocorrelation varies across the study area. High Gi* values indicate "hot spots" of high newborn care practices, while low Gi* values indicate "cold spots”.^24^

### Statistical analysis

The authors used descriptive statistics to summarize sociodemographic characteristics and explored preliminary associations through bivariate analyses. To account for the hierarchical data structure, a Multilevel Logistic Regression Model (MLRM) was employed, incorporating both fixed effects (to calculate odds ratios) and random effects (assessed through the intraclass correlation coefficient, ICC). The model-building process was sequential, beginning with a null model to assess baseline variability, followed by models incorporating sociodemographic characteristics, and culminating in a comprehensive final model. These analyses were conducted in STATA version 16, with sample weights applied to ensure representativeness, and statistical significance set at a p-value < 0.05, and model fit assessed by deviance. The geospatial analysis was conducted in R version 4.3.2 to examine the geographical distribution of newborn care practices. Different R packages, including “sf”, “spdep”, “tmap”, “ggplot2”, and “raster”, were used for spatial analysis and visualization.^25^

## Results

The accompanying pie chart illustrates the prevalence of early newborn care in Somalia, indicating that a significant majority of the population (94.77%) lacks access to early newborn care services, while only 5.23% of the population benefits from such care. The frequency distribution of the participants by socio-demographic and health characteristics is presented in Table 1. Findings reveal that 56.5% of the study population consisted of women aged 15-29 years, indicating a young demographic. The majority of participants were married (91.9%), with a significant number residing in urban (38.2%) or nomadic (35.4%) settings. A concerning trend was observed in educational attainment, with 84.3% of the women having never attended school, a situation mirrored by their mothers. Employment opportunities were scarce, with 98.9% reporting no work experience. Economic disparities were evident, as 45.7% of households were classified as poor, while only 34.8% were deemed rich. Access to media was limited; over 90% of participants did not engage with radio (91.5%) or television (90.2%) at least weekly. Maternal and child health indicators reflected high fertility rates, with over half of the births being of the fourth order or higher (52.0%). Notably, a significant majority (73.4%) perceived access to healthcare as a "big problem," with 80.2% of births occurring at home or non-facility locations, highlighting critical barriers to healthcare access.

**Table 1:** Socio-demographic and health characteristics of early newborn care in Somalia (N = 15,024).

**Variables**	**Categories**	**Frequency (n)**	**Percentage (%)**
Region	Awdal	624	4.15
	Woqooyi Galbeed	1,032	6.87
	Togdheer	1,020	6.79
	Sool	1,186	7.89
	Sanaag	1,245	8.29
	Bari	903	6.01
	Nugaal	897	5.97
	Mudug	842	5.60
	Galgaduud	804	5.35
	Hiraan	729	4.85
	Middle Shabelle	791	5.26
	Banadir	1,554	10.34
	Bay	350	2.33
	Bakool	1,076	7.16
	Gedo	1,005	6.69
	Lower Juba	966	6.43
Residence	Rural	3,970	26.42
	Urban	5,738	38.19
	Nomadic	5,316	35.38
Ever Attended School	Yes	2,355	15.67
	No	12,669	84.33
Maternal Education	No Education	12,666	84.31
	Primary	1,787	11.89
	Secondary	445	2.96
	Higher	126	0.84
Frequency of Listening to Radio	At least once a week	895	5.96
	Less than once a week	388	2.58
	Not at all	13,741	91.46
Frequency of Watching Television	At least once a week	1,133	7.54
	Less than once a week	344	2.29
	Not at all	13,547	90.17
Internet Usage	Yes	1,394	9.28
	No	13,630	90.72
Current Marital Status	Married	13,810	91.92
	Divorced	836	5.56
	Widowed	378	2.52
Birth Type	Single	14,812	98.59
	Multiple	212	1.41
Sex of Child	Male	7,917	52.70
	Female	7,107	47.30
Wealth Index	Poor	6,859	45.65
	Middle	2,932	19.52
	Rich	5,233	34.83
Maternal Age	15-29 Years	8,483	56.46
	30-39 Years	5,510	36.67
	40-49 Years	1,031	6.86
Birth Order	1-2	4,913	32.70
	3	2,296	15.28
	4+	7,815	52.02
Women's Occupation	Not Worked	14,859	98.90
	Worked	165	1.10
Barriers to Accessing Care	Not a big problem	3,990	26.56
	Big problem	11,034	73.44
Place of Delivery	Health Facility	2,973	19.79
	Home and Others	12,051	80.21
Household Size	1 Member	545	3.63
	2 Members	1,152	7.67
	3-5 Members	6,290	41.87
	More than 5 Members	7,037	46.84
Number of Children	0-1	949	6.32
	2-3	4,264	28.38
	4-5	4,326	28.79
	6-7	3,027	20.15
	8-9	1,614	10.74
	10+	844	5.62

The results of bivariate analysis are presented in Table 2, and findings reveal several statistically significant associations with demographic, socioeconomic, and health-related variables. Notably, a significant relationship was found between region and the dependent variable, with substantial disparities observed ($\chi^{2}$ = 126.68, p < 0.001). For instance, participants from Awdal reported only 8.81% affirming the dependent variable, while regions like Togdheer (10.00%) and Woqooyi Galbeed (7.75%) displayed similar patterns, indicating regional variations in responses. Residence type showed no significant association ($\chi^{2}$ = 3.67, p = 0.159), as the proportions remained relatively consistent across rural (5.06%), urban (5.68%), and nomadic (5.98%) categories. However, educational attainment exhibited a strong link ($\chi^{2}$ = 124.48, p < 0.001), with those who attended school showing a higher affirmative response (10.49%) compared to those who did not (4.72%).

**Table 2:** Bivariate analysis of determinants of early newborn care in Somalia.

**Variables**	**Categories**	**Yes (n) (%)**	**No (n) (%)**	**p-value of**$\chi^{2}$
Region	Awdal	55 (8.81)	569 (91.19)	<0.001
	Woqooyi Galbeed	80 (7.75)	952 (92.25)	
	Togdheer	102 (10.00)	918 (90.00)	
	Sool	75 (6.32)	1,111 (93.68)	
	Sanaag	83 (6.67)	1,162 (93.33)	
	Bari	33 (3.65)	870 (96.35)	
	Nugaal	55 (6.13)	842 (93.87)	
	Mudug	21 (2.49)	821 (97.51)	
	Galgaduud	26 (3.23)	778 (96.77)	
	Hiraan	39 (5.35)	690 (94.65)	
	Middle Shabelle	51 (6.45)	740 (93.55)	
	Banadir	81 (5.21)	1,473 (94.79)	
	Bay	11 (3.14)	339 (96.86)	
	Bakool	72 (6.69)	1,004 (93.31)	
	Gedo	23 (2.29)	982 (97.71)	
	Lower Juba	38 (3.93)	928 (96.07)	
Residence	Rural	201 (5.06)	3,769 (94.94)	0.159
	Urban	326 (5.68)	5,412 (94.32)	
	Nomadic	318 (5.98)	4,998 (94.02)	
Ever Attended School	Yes	247 (10.49)	2,108 (89.51)	<0.001
	No	598 (4.72)	12,071 (95.28)	
Maternal Education	No Education	598 (4.72)	12,068 (95.28)	<0.001
	Primary	161 (9.01)	1,626 (90.99)	
	Secondary	59 (13.26)	386 (86.74)	
	Higher	27 (21.43)	99 (78.57)	
Frequency of Listening to Radio	At least once a week	83 (9.27)	812 (90.73)	<0.001
	Less than once a week	51 (13.14)	337 (86.86)	
	Not at all	711 (5.17)	13,030 (94.83)	
Frequency of Watching Television	At least once a week	144 (12.71)	989 (87.29)	<0.001
	Less than once a week	47 (13.66)	297 (86.34)	
	Not at all	654 (4.83)	12,893 (95.17)	
Internet Usage	Yes	176 (12.63)	1,218 (87.37)	<0.001
	No	669 (4.91)	12,961 (95.09)	
Current Marital Status	Married	750 (5.43)	13,060 (94.57)	<0.001
	Divorced	77 (9.21)	759 (90.79)	
	Widowed	18 (4.76)	360 (95.24)	
Birth Type	Single	843 (5.69)	13,969 (94.31)	0.003
	Multiple	2 (0.94)	210 (99.06)	
Sex of Child	Male	448 (5.66)	7,469 (94.34)	0.847
	Female	397 (5.59)	6,710 (94.41)	
Wealth Index	Poor	169 (2.46)	6,690 (97.54)	<0.001
	Middle	182 (6.21)	2,750 (93.79)	
	Rich	494 (9.44)	4,739 (90.56)	
Maternal Age	15-29 Years	510 (6.01)	7,973 (93.99)	0.062
	30-39 Years	281 (5.10)	5,229 (94.90)	
	40-49 Years	54 (5.24)	977 (94.76)	
Birth Order	1-2	296 (6.02)	4,617 (93.98)	0.280
	3	119 (5.18)	2,177 (94.82)	
	4+	430 (5.50)	7,385 (94.50)	
Women's Occupation	Not Worked	830 (5.59)	14,029 (94.41)	0.052
	Worked	15 (9.09)	150 (90.91)	
Barriers to Accessing Care	Not a big problem	292 (7.32)	3,698 (92.68)	<0.001
	Big problem	553 (5.01)	10,481 (94.99)	
Place of Delivery	Health Facility	486 (16.35)	2,487 (83.65)	<0.001
	Home and Others	359 (2.98)	11,692 (97.02)	
Household Size	1 Member	32 (5.87)	513 (94.13)	0.830
	2 Members	61 (5.30)	1,091 (94.70)	
	3-5 Members	365 (5.80)	5,925 (94.20)	
	6 or more Members	387 (5.50)	6,650 (94.50)	
Number of Children	0-1	158 (16.65)	791 (83.35)	<0.001
	2-3	261 (6.12)	4,003 (93.88)	
	4-5	181 (4.18)	4,145 (95.82)	
	6-7	128 (4.23)	2,899 (95.77)	
	8-9	78 (4.83)	1,536 (95.17)	
	10+	39 (4.62)	844 (95.38)	

Maternal education level also indicated significant disparities. Women with higher education displayed the highest affirmative responses (21.43%), while those with no education had the lowest (4.72%). Furthermore, regular media exposure was significantly correlated with the dependent variable, as those who listened to the radio at least once a week had a higher affirmation rate (9.27%) compared to non-listeners (5.17%). Interestingly, internet usage was associated with a higher proportion of affirmative responses (12.63%) compared to non-users (4.91%). Current marital status also showed significant results, with divorced women (9.21%) reporting a higher proportion compared to married individuals (5.43%).

Birth type also revealed significant differences, with single births reflecting a higher proportion of affirmative responses (5.69%) compared to multiple births (0.94%). The wealth index was significantly associated with ENC, indicating that a higher prevalence of ENC was observed among wealthier households (9.44%) compared to poorer households (2.46%). Maternal age did not reveal a significant correlation, nor did birth order. Interestingly, women’s occupation approached significance, with those who worked reporting a higher proportion of affirmatives (9.09%). Barriers to accessing care and the place of delivery were significantly associated with ENC. Household size and the number of children born did not show significant associations, although the latter indicated some trends.

The findings of a multilevel, multivariable logistic regression analysis are presented in Table 3, identifying the individual, household, and community-level determinants of early newborn care in Somalia. The sequential modeling approach allows for a nuanced understanding of these factors, culminating in the fully adjusted final model (Model III).

**Table 3:** Multilevel logistic regression analysis of factors associated with ENC in Somalia.

**Variables**	**Categories**	**Null Model (Model 0)**	**Model I (AOR, 95% CI)**	**Model II (AOR, 95% CI)**	**Model III (AOR, 95% CI)**
Region	Awdal			Reference	Reference
	Woqooyi Galbeed			1.04 (0.68-1.60)	1.04 (0.67-1.61)
	Togdheer			1.13 (0.75-1.72)	1.07 (0.70-1.65)
	Sool			0.64 (0.41-1.00)*	0.59 (0.37-0.93)*
	Sanaag			0.69 (0.45-1.06)	0.63 (0.40-0.98)*
	Bari			0.61 (0.37-1.03)	0.67 (0.40-1.14)
	Nugaal			1.12 (0.71-1.77)	1.22 (0.77-1.95)
	Mudug			0.42 (0.24-0.75)*	0.48 (0.26-0.86)*
	Galgaduud			0.47 (0.27-0.81)*	0.52 (0.30-0.91)*
	Hiraan			0.94 (0.57-1.55)	0.95 (0.57-1.58)
	Middle Shabelle			0.78 (0.48-1.26)	0.76 (0.46-1.24)
	Banadir			0.65 (0.42-1.00)*	0.62 (0.40-0.96)*
	Bay			0.30 (0.14-0.64)*	0.30 (0.14-0.64)*
	Bakool			0.90 (0.58-1.39)	0.96 (0.61-1.52)
	Gedo			0.49 (0.28-0.87)*	0.62 (0.35-1.11)
	Lower Juba			0.61 (0.37-0.98)*	0.54 (0.32-0.89)*
Residence	Rural			Reference	Reference
	Urban			1.28 (1.02-1.60)*	1.26 (1.00-1.59)*
	Nomadic			1.16 (0.93-1.45)	1.10 (0.87-1.38)
Maternal Education	No Education		Reference		Reference
	Primary		1.25 (1.02-1.54)*		1.06 (0.86-1.32)
	Secondary		1.25 (0.89-1.76)		0.94 (0.66-1.34)
	Higher		1.62 (0.97-2.72)		1.07 (0.63-1.82)
Frequency of Listening to the Radio	At least once a week		Reference		Reference
	Less than once a week		1.44 (0.95-2.20)		1.49 (0.97-2.30)
	Not at all		0.91 (0.69-1.19)		0.86 (0.65-1.14)
Frequency of Watching Television	At least once a week		Reference		Reference
	Less than once a week		1.00 (0.67-1.50)		1.16 (0.76-1.76)
	Not at all		0.70 (0.55-0.89)*		0.90 (0.70-1.15)
Internet Usage	Yes		Reference		Reference
	No		0.89 (0.70-1.13)		0.90 (0.70-1.15)
Current Marital Status	Married		Reference		Reference
	Divorced		1.13 (0.87-1.48)		1.08 (0.82-1.43)
	Widowed		0.87 (0.53-1.44)		0.85 (0.51-1.41)
Birth Type	Single		Reference		Reference
	Multiple		0.20 (0.05-0.82)*		0.23 (0.06-0.95)*
Sex of Child	Male		Reference		Reference
	Female		0.91 (0.79-1.06)		0.95 (0.82-1.10)
Wealth Index	Poor		Reference		Reference
	Middle		2.48 (1.96-3.13)*		1.92 (1.50-2.45)*
	Rich		3.20 (2.57-3.98)*		2.02 (1.59-2.57)*
Maternal Age (Years)	15-29		Reference		Reference
	30-39		1.04 (0.86-1.25)		0.97 (0.80-1.18)
	40-49		1.24 (0.89-1.73)		1.14 (0.81-1.62)
Birth Order	1-2		Reference		Reference
	3		2.37 (1.83-3.07)*		2.51 (1.92-3.27)*
	4+		14.59 (9.65-22.06)*		16.08 (10.43-24.77)*
Women’s Occupation	Not Worked		Reference		Reference
	Worked		1.30 (0.74-2.31)		1.66 (0.93-2.99)
Barriers to Accessing Care	Not a big problem			Reference	Reference
	Big problem			0.84 (0.72-0.98)*	0.94 (0.80-1.11)
Place of Delivery	Health Facility			Reference	Reference
	Home and Others			0.17 (0.15-0.20)*	0.22 (0.18-0.26)*
Household Size	1 Member		Reference		Reference
	2 Members		0.90 (0.56-1.43)		0.98 (0.61-1.58)
	3-5 Members		0.96 (0.65-1.42)		0.98 (0.65-1.46)
	More than 5 Members		0.90 (0.61-1.34)		0.95 (0.63-1.41)
Number of Children	0-1		Reference		Reference
	2-3		0.26 (0.20-0.34)*		0.29 (0.22-0.37)*
	4-5		0.03 (0.02-0.05)*		0.03 (0.02-0.05)*
	6-7		0.02 (0.01-0.03)*		0.02 (0.01-0.04)*
	8-9		0.02 (0.01-0.03)*		0.02 (0.01-0.04)*
	10+		0.02 (0.01-0.03)*		0.02 (0.01-0.04)*

The analysis reveals that several factors at different levels are significantly associated with the receipt of early newborn care, even after adjusting for potential confounders in the final model. The findings of the Model III depict significant regional disparities were a prominent finding; compared to the Awdal region, newborns in Bay (AOR = 0.96, 95% CI: 0.61-1.52), Mudug (AOR = 0.48, 95% CI: 0.26-0.86), Galgaduud (AOR = 0.52, 95% CI: 0.30-0.91), and Lower Juba (AOR = 0.54, 95% CI: 0.32-0.89), among others, had substantially lower odds of receiving early care. At the household level, a strong socio-economic gradient was observed. Newborns from the richest households were twice as likely to receive early care as those from the poorest households (AOR = 2.02, 95% CI: 1.59-2.57). Crucially, obstetric factors were powerful predictors. The place of delivery was profoundly significant, with newborns delivered at home having 78% lower odds of receiving care compared to those delivered in a health facility (AOR = 0.22, 95% CI: 0.18-0.26). A paradoxical relationship emerged with parity; newborns of higher birth order (4+) were significantly more likely to receive care (AOR = 16.08, 95% CI: 10.43-24.77). Conversely, an increase in the total number of living children in the household was associated with drastically lower odds of the newborn receiving care. Additionally, newborns from multiple births were significantly less likely to receive care than singletons (AOR = 0.23, 95% CI: 0.06-0.95). It is noteworthy that the effects of some variables were attenuated in the final model. For instance, maternal primary education, which was a significant predictor in Model I, lost its significance in Model III. This suggests its influence may be mediated by factors such as wealth and place of delivery. Similarly, the initial significant association between a perceived "big problem" in accessing care and lower odds of receiving care (Model II) became non-significant after full adjustment in Model III.

### Model variation and fit statistics

The measures of variation and model fit statistics for the four multilevel logistic regression models are presented in Table 4. These diagnostics were used to justify the use of a multilevel approach and to assess the explanatory power and comparative fit of the successive models. The null model (Model 0), which included no predictor variables, was first established to partition the total variance in early newborn care into between-community (regional) and within-community components. The random-effects variance ($\tau^{2}$) at the regional level was 0.776 and was highly significant (p < 0.001). The Intra-Class Correlation Coefficient (ICC) for the null model was 0.191, indicating that 19.1% of the total variation in the receipt of early newborn care is attributable to differences between regions. This substantial clustering effect confirms the appropriateness of the multilevel modeling strategy, as standard logistic regression would violate the assumption of independent observations and yield biased standard errors.

**Table 4:** Measurement of variation and model fit statistics.

**Measurement of Variation**	**Null Model**	**Model I**	**Model II**	**Model III**
**Random Effects**				
$\tau^{2}$ (Variance)	0.776	0.248	0.314	0.268
p-value	< 0.001	< 0.001	< 0.001	< 0.001
ICC	0.191	0.070	0.087	0.075
LR test	146.00	27.69	47.11	31.95
**Model Fit Statistics**				
AIC	6363.49	5813.97	5809.59	5466.53
BIC	6378.73	6034.85	5969.55	5832.13
Deviance (-2Log-likelihood)	6359.49	5755.97	5767.59	5370.53

As predictors were sequentially introduced in Models I, II, and III, a progressive reduction in the between-region variance was observed. The variance component decreased from 0.776 in the null model to 0.268 in the final, fully adjusted model (Model III). This demonstrates that the individual, household, and community-level variables included in the analysis successfully explained a significant portion of the regional variation. Specifically, the predictors in the final model accounted for approximately 65.5% of the variance between regions observed in the null model. Despite this reduction, the remaining regional-level variance in Model III remained statistically significant (p < 0.001), suggesting that other unmeasured contextual factors continue to contribute to regional disparities in early newborn care. The model fit was assessed using the Likelihood Ratio (LR) test, Akaike Information Criterion (AIC), and Bayesian Information Criterion (BIC). The LR test was significant at each stage, confirming that each successive model provided a statistically significant improvement in fit over the preceding one. Furthermore, the values for both AIC and BIC consistently decreased with the addition of variables, with Model III exhibiting the lowest values (AIC = 5466.53; BIC = 5832.13). This indicates that the final, fully-adjusted model (Model III), which incorporates individual, household, and community-level factors, represents the most parsimonious and best-fitting model for explaining the determinants of early newborn care in the Somali context.

### Spatial distribution of newborn care in Somalia

**Figure 1:**
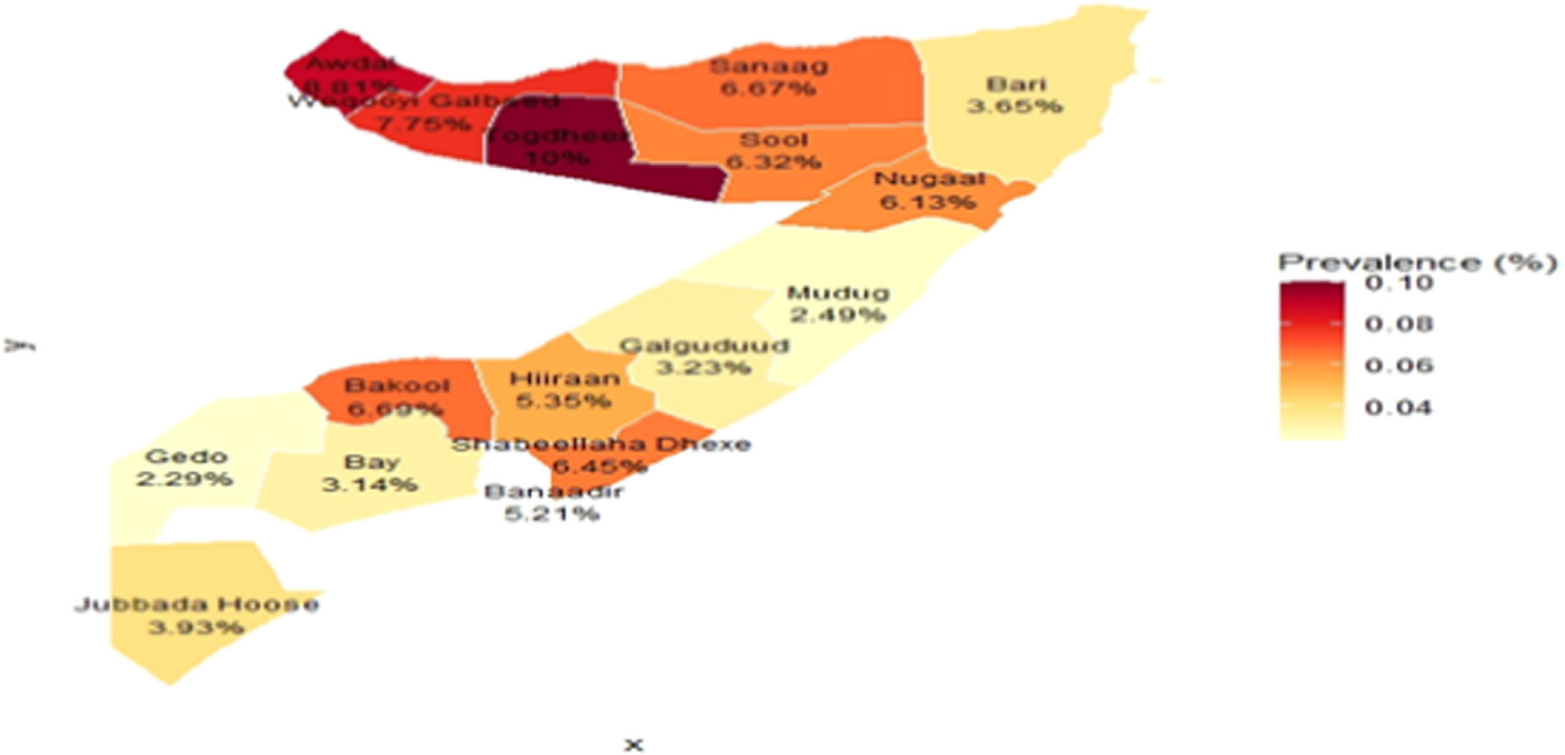
Spatial distribution of immediate care of newborns in Somalia.

The spatial analysis of immediate newborn care in Somalia, illustrated in Figure 1, reveals profound regional disparities and critically low coverage nationwide, based on the 2020 SDHS data. A distinct geographical pattern emerges, with the highest prevalence of care concentrated in the northwestern regions of Awdal, Woqooyi Galbeed, and Togdheer, where the proportion of newborns receiving care reached approximately 10%. In stark contrast, the lowest coverage rates, around 2%, were widespread across the rest of the country. These poorly performing areas include the northeastern regions of Bari and Sanaag and extend through the central and southern territories, including Galguduud, Mudug, Hiraan, Shabeellaha Dhexe, Bay, Gedo, Jubbada Hoose, and notably, the capital region of Banaadir. This pronounced geographical gradient, particularly the disparity between the northwest and other regions, suggests that variations in health system infrastructure, regional governance, and security likely play a significant role in care delivery. Critically, even the highest observed rate of 10% highlights a nationwide public health failure, indicating that the vast majority of Somali newborns are missing out on essential, life-saving interventions at birth.

### *Global Moran*I*analysis (spatial and incremental autocorrelation of newborn care)*

**Figure 2:**
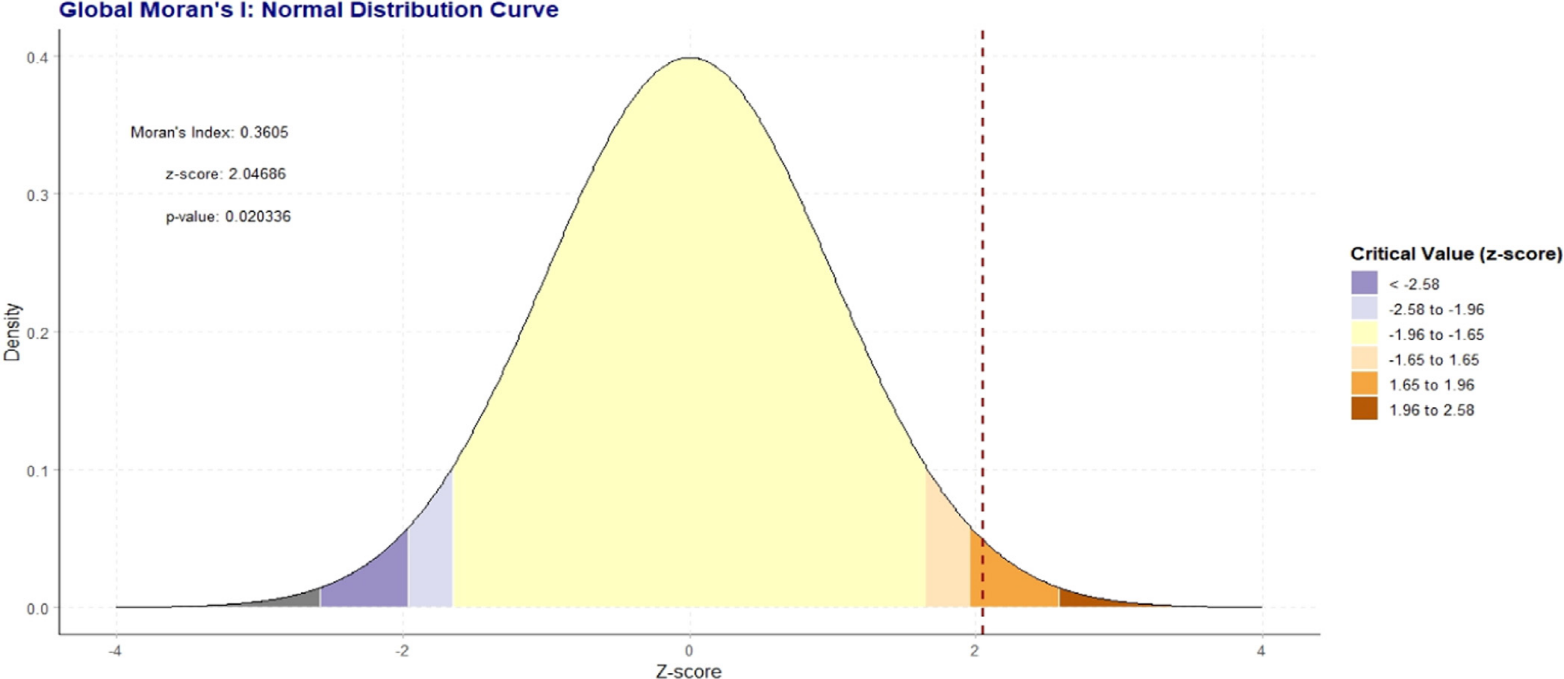
Spatial autocorrelation analysis of early newborn care practice in Somalia.

The Global Moran's I analysis confirms that the spatial distribution of the variable is significantly clustered and not the result of a random process. The positive Moran's Index of 0.36 indicates a tendency for regions with similar values to be located near one another, forming distinct geographic groupings. This visual pattern is statistically validated by a z-score of 2.05 and a p-value of 0.02. Because this p value is below the standard 0.05 threshold for statistical significance, the null hypothesis of complete spatial randomness is rejected, providing strong evidence that the observed clustering is a real and meaningful geographic phenomenon (Figure 2).

**Figure 3:**
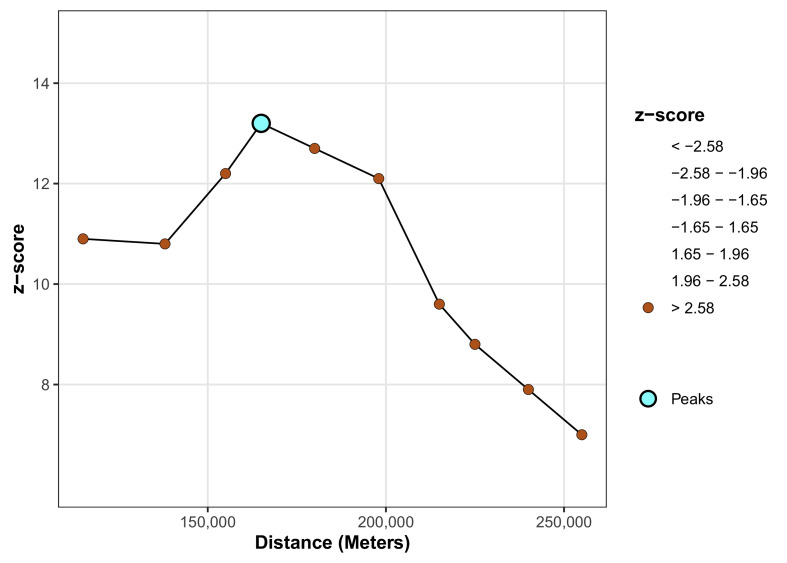
Incremental autocorrelation of early newborn care practice in Somalia.

To identify the specific distance at which this clustering is most pronounced, an incremental spatial autocorrelation analysis was performed. This analysis reveals a distinct peak in the Z-score at a distance of approximately 166,000 meters (166 km). At this distance, the Zscore reaches its maximum value of over 13, indicating an extremely intense and highly significant clustering effect (p < 0.001). This result pinpoints 166 km as the characteristic scale of the spatial process, making it the optimal distance parameter for use in further local analyses, such as identifying the precise locations of hot and cold spots (Figure 3).

### Local Moran’s I and significance

**Figure 4:**
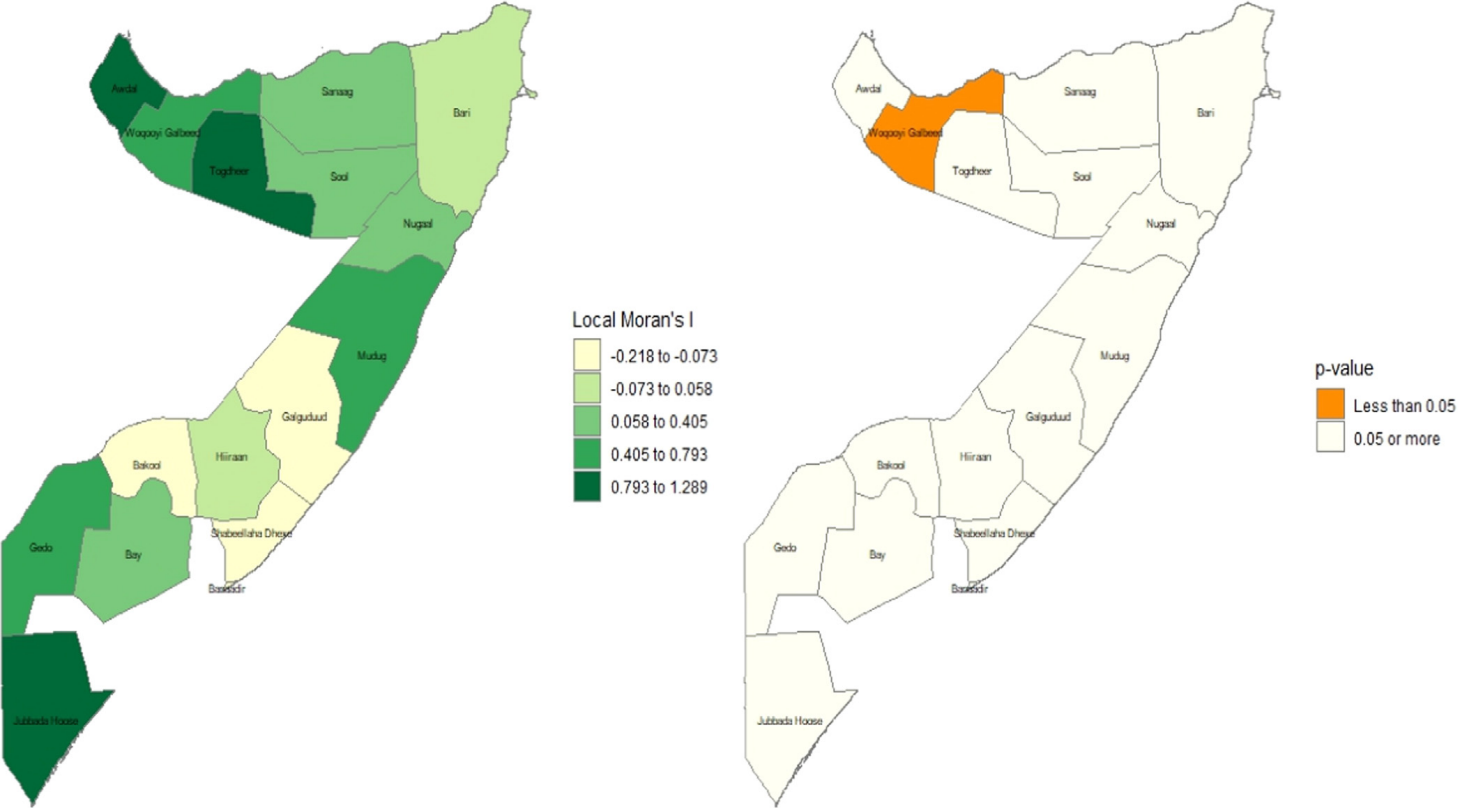
Local Moran’s I and its p-value for significance.

A Local Indicators of Spatial Association (LISA) analysis was conducted to identify significant spatial clusters of newborn care practices across the regions of Somalia, and the findings are illustrated in Figure 4. The analysis revealed a single, statistically significant "hot spot" in the northwestern region of Woqooyi Galbeed, which exhibited a high positive Local Moran's I value (in the 0.793 to 1.289 range) and a p-value of less than 0.05. This result indicates a significantly high cluster, where neighbors with similarly high uptake surround a region with a high uptake of recommended newborn care practices. While other regions, such as Togdheer and Jubbada Hoose, also showed high positive Moran's I values, these patterns were not statistically significant (p ≥ 0.05). Consequently, the evidence suggests a highly localized geographical clustering of favorable newborn care practices in the northwest, whereas the distribution of these practices elsewhere in the country appears to be spatially random at this regional scale.

### Getis-Ord Gi* and significance

**Figure 5:**
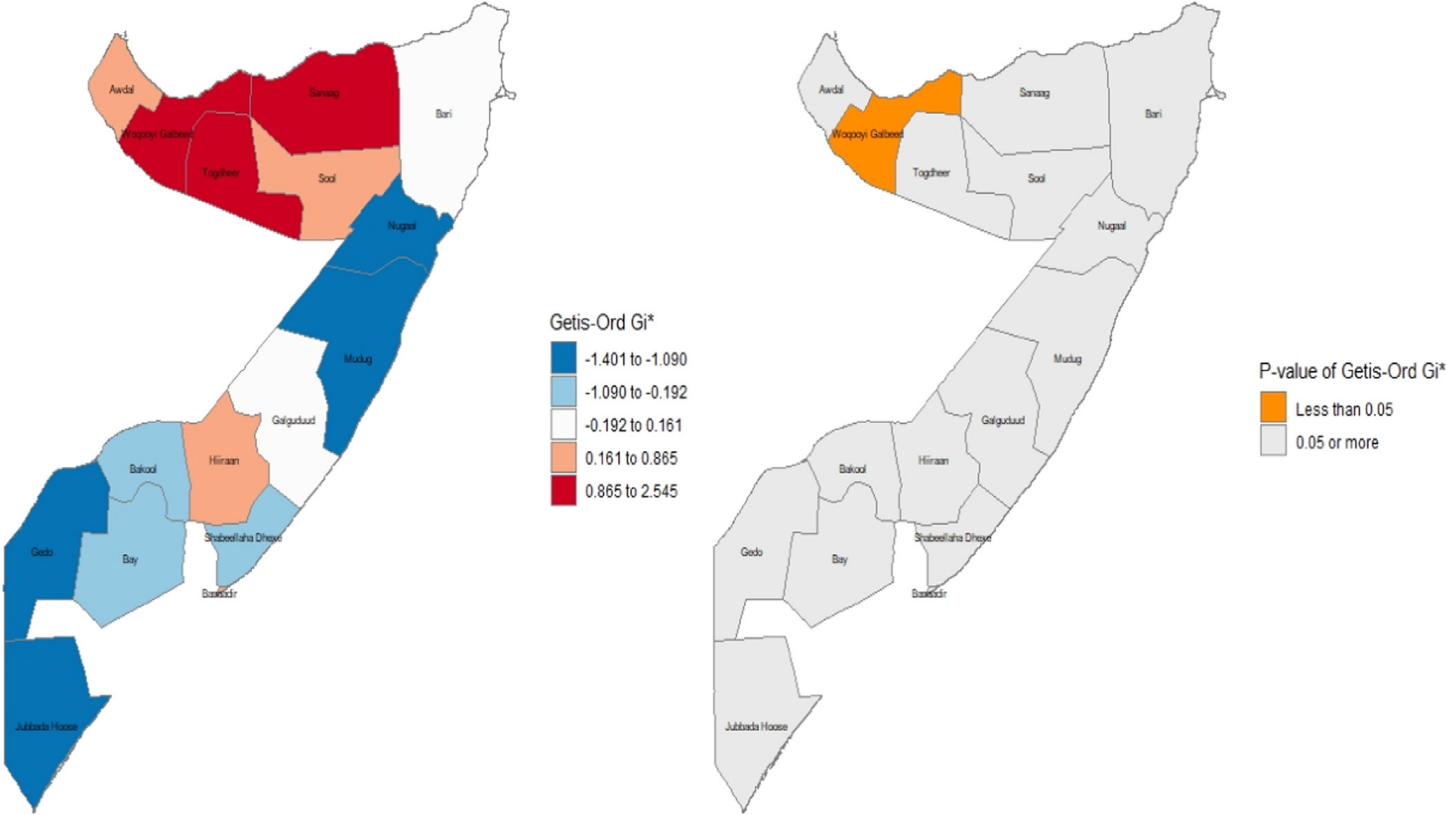
Getis-Ord Gi* I and its p-value for significance.

The results of Getis-Ord Gi* I and its p-value for significance are displayed in Figure 5. The findings of the Getis-Ord Gi* analysis reveal a striking dichotomy between the spatial patterns of high- and low-performing areas. The analysis identified a single, robust, and statistically significant hot spot of favorable newborn care practices (p < 0.05) concentrated in the northwestern region of Woqooyi Galbeed. In contrast, no statistically significant cold spots were found, despite visually apparent clusters of poor practices in the southern (Gedo, Jubbada Hoose) and northeastern (Mudug) regions. This asymmetry is a compelling result, suggesting that while positive health practices have achieved a significant, localized concentration of excellence, the underlying drivers of poor practices are more spatially diffuse and generalized. This null finding for cold spots is critically important, as it implies that the challenges in these regions are not isolated but may reflect systemic issues. Programmatically, this suggests that interventions must be twofold: learning from the localized success of the Woqooyi Galbeed hot spot while implementing broad, systemic health improvements to address the widespread, non-clustered challenges elsewhere.

## Discussion

This study presents the first comprehensive national-level analysis of the determinants and geographic distribution of early newborn care (ENC) in Somalia, utilizing recent data from the 2020 Somali Health and Demographic Survey. The findings reveal a critically low prevalence of adequate ENC, with only 5.23% of newborns receiving the complete package of care within the first two days of life. This alarmingly low figure underscores a profound public health crisis and highlights the immense challenges facing Somalia’s fragile health system. The multilevel and spatial analyses identified powerful determinants at the individual, household, and community levels, revealing deep-seated inequities that drive this poor outcome. The key predictors included place of delivery, household wealth, geographic region, birth type, and a complex interplay between birth order and the number of living children. Findings depict that newborns delivered in a health facility were substantially more likely to receive ENC compared to those born at home, a finding consistent with a vast body of literature from other resource-limited settings, including neighboring Ethiopia^2^,^7^,^8^ and Kenya.^10^ With over 80% of births in Somalia occurring at home, this factor represents the single most significant barrier to improving newborn survival. It highlights a dual challenge: the lack of physical access to or trust in the formal health system and the absence of robust community-based strategies to deliver essential services to mothers and newborns at home. Similarly, the strong dose-response relationship between household wealth and ENC uptake mirrors findings globally^24^ and regionally.^2^,^6^ The stark reality that newborns from the richest quintile are over twice as likely to receive care as those from the poorest demonstrates that poverty is a fundamental barrier, limiting a family’s ability to overcome costs associated with transportation, services, and opportunity costs of seeking care.

The spatial analysis provided crucial, actionable insights by mapping the geographic inequalities in ENC. The identification of a statistically significant hot spot of relatively higher care in the northwestern regions (Awdal, Woqooyi Galbeed, Togdheer) and cold spots of extremely low care in the south and the capital region of Banadir corresponds with the known political, security, and developmental disparities within Somalia.^4^,^17^ The northwest regions, which have experienced relative stability and have more established governance structures, likely possess a more functional health infrastructure. Conversely, the southern cold spots and the low-performing Banadir region are areas more acutely affected by conflict, displacement, and systemic fragmentation, severely hampering health service delivery.^4^ This geographic evidence moves beyond general assumptions and provides policymakers with a clear map of priority zones for intensive intervention. One of the most nuanced findings from this study is the paradoxical relationship with parity. While a higher birth order was associated with a dramatically increased likelihood of receiving ENC, a higher number of living children was associated with a drastically lower likelihood. The former may suggest that more experienced mothers are better able to navigate the health system or have greater awareness of the importance of ENC. However, the latter finding likely points to a "resource dilution" effect, where households with many children face greater economic and time constraints, making it difficult to prioritize care for the newest child. This complex interplay has not been widely reported with such stark contrasts and represents a significant contribution of this paper, suggesting that interventions must be tailored differently for primiparous mothers versus multiparous mothers with large families. Furthermore, the finding that multiple-birth newborns are significantly less likely to receive care is a critical alert. These newborns are inherently at higher risk and require more specialized attention; yet, the present results show they are being left behind, likely due to the increased complexity and cost of their care, which overwhelm both families and under-resourced health facilities.^10^

In contrast to some studies, where maternal education remains a strong, independent predictor of health-seeking behavior.^2^,^20^ The final model showed that its effect was attenuated to non-significance after controlling for wealth and place of delivery. This suggests that in the extreme context of Somalia, the structural barriers of poverty and lack of physical access to health facilities are so overwhelming that they largely eclipse the potential benefits of maternal education. A mother may know what is best for her child, but lack the financial means or physical access to act on that knowledge. This underscores that interventions focused solely on health education, without concurrently addressing economic and structural barriers, are unlikely to succeed.

### Strengths and limitations of the study

This research has several strengths, including mapping hot and cold spots, which provides tangible evidence for geographically targeted resource allocation. The findings directly inform progress, or lack thereof, towards Sustainable Development Goal 3 (Good Health and Well-being), particularly Target 3.2, which aims to end preventable deaths of newborns and children under five. However, this study has a few limitations. Its cross-sectional design precludes the establishment of causality. The data are based on maternal self-report, which may be subject to recall bias. Furthermore, the survey could not be conducted in several insecure areas, potentially leading to an underestimation of the severity of the problem in the most vulnerable zones. The composite nature of the outcome variable, while useful for an overall assessment, does not allow for differentiation of which specific ENC components are most neglected. The outcome distribution is not balanced in this study; a further study may consider employing balancing techniques to obtain more insightful findings.

## Conclusion

Early newborn care in Somalia is in a state of crisis, defined by critically low coverage and stark geographic and socioeconomic disparities. The path forward requires a multi-pronged strategy that prioritizes strengthening health systems in the identified cold spots, promoting facility-based deliveries by making them affordable and accessible, and designing targeted social protection programs for the poorest households. Special attention must be given to high-risk groups, including mothers with multiple births and those with large families. Urgent, evidence-based, and geographically-focused interventions are essential to achieve the goal of ending preventable newborn deaths in Somalia.

## Authors’ contributions

Conceptualization: Abdirahman Omer Ali, Abdisalam Mahdi Hassan, Nura Mohamed Omer. Methodology: Abdirahman Omer Ali, Muhyadin Yusuf Dahir, Awo Mohamed Kahie, Md Moyazzem Hossain. Software: Awo Mohamed Kahie. Validation: Abdirahman Omer Ali, Muhyadin Yusuf Dahir, Md. Moyazzem Hossain. Formal analysis: Abdirahman Omer Ali, Abdisalam Mahdi Hassan. Visualization: Abdirahman Omer Ali, Abdisalam Mahdi Hassan, Md. Moyazzem Hossain Data curation: Hodo Abdi Abdilahi, Abdirahman Omer Ali, Nura Mohamed Omer. Writing—original draft preparation: Abdirahman Omer Ali, Hodo Abdi Abdilahi, Awo Mohamed Kahie. Review and editing: Yusuf Abdi Hared, Md. Moyazzem Hossain. Supervision: Abdirahman Omer Ali, Md. Moyazzem Hossain. All authors have read and approved the final version of the manuscript.

## Ethics approval and consent to participate

The study utilized publicly available, de-identified data from the Somali Demographic and Health Survey (SDHS). The SHDS obtained ethical clearance from the relevant ethical review boards in Somalia. As such, this study did not require additional ethical clearance.

## Consent for publication

Not applicable.

## Data availability statement

The datasets analyzed during the current study are available in the Demographic and Health Surveys (DHS) Program repository, https://dhsprogram.com/data/available-datasets.cfm. Specifically, the 2020 Somali Health and Demographic Survey data were used.

## Funding

This research received no specific grant from any funding agency in the public, commercial, or not-for-profit sectors.

## Conflicts of interest

The authors declare no conflicts of interest.

## Appendix Supplementary materials

Image, application 1

## Footnotes

Supplementary material associated with this article can be found in the online version at doi:10.1016/j.jped.2025.101496.
