# Supplementary material for: Determinants and spatial distribution of early newborn care in Somalia: evidence from the 2020 Somalia health and demographic survey
Source: J Pediatr (Rio J). 2026 Jan 6;102(2):101496. doi: 10.1016/j.jped.2025.101496 (PMC12809074; doi:10.1016/j.jped.2025.101496)
Supplement: Supplementary file 1 [file mmc1.docx]

**JPED-D-25-00342_ Supplementary Material**

**Supplementary Table 1** List of covariates used in this study.

| **Variables** | **Categories** |
| --- | --- |
| Region | Awdal |
|  | Woqooyi Galbeed |
|  | Togdheer |
|  | Sool |
|  | Sanaag |
|  | Bari |
|  | Nugaal |
|  | Mudug |
|  | Galgaduud |
|  | Hiraan |
|  | Middle Shabelle |
|  | Banadir |
|  | Bay |
|  | Bakool |
|  | Gedo |
|  | Lower Juba |
| Residence | Rural |
|  | Urban |
|  | Nomadic |
| Ever Attended School | Yes |
|  | No |
| Maternal Education | No Education |
|  | Primary |
|  | Secondary |
|  | Higher |
| Frequency of Listening to Radio | At least once a week |
|  | Less than once a week |
|  | Not at all |
| Frequency of Watching Television | At least once a week |
|  | Less than once a week |
|  | Not at all |
| Internet Usage | Yes |
|  | No |
| Current Marital Status | Married |
|  | Divorced |
|  | Widowed |
| Birth Type | Single |
|  | Multiple |
| Sex of Child | Male |
|  | Female |
| Wealth Index | Poor |
|  | Middle |
|  | Rich |
| Maternal Age | 15-29 Years |
|  | 30-39 Years |
|  | 40-49 Years |
| Birth Order | 1-2 |
|  | 3 |
|  | 4+ |
| Women's Occupation | Not Worked |
|  | Worked |
| Barriers to Accessing Care | Not a big problem |
|  | Big problem |
| Place of Delivery | Health Facility |
|  | Home and Others |
| Household Size | 1 Member |
|  | 2 Members |
|  | 3-5 Members |
|  | More than 5 Members |
| Number of Children | 0-1 |
|  | 2-3 |
|  | 4-5 |
|  | 6-7 |
|  | 8-9 |
|  | 10+ |
